# Bistability and up/down state alternations in inhibition-dominated randomly connected networks of LIF neurons

**DOI:** 10.1038/s41598-017-12033-y

**Published:** 2017-09-20

**Authors:** Elisa M. Tartaglia, Nicolas Brunel

**Affiliations:** 1 Sorbonne University, UPMC University of Paris 06, INSERM, CNRS, Vision Institute, F-75012 Paris, France; 2 Department of Neurobiology, The University of Chicago, Chicago, Illinois USA; 3 Department of Statistics, The University of Chicago, Chicago, Illinois USA; 4 Department of Neurobiology, Duke University, NC, USA; 5 Department of Physics, Duke University, NC, USA

## Abstract

Electrophysiological recordings in cortex *in vivo* have revealed a rich variety of dynamical regimes ranging from irregular asynchronous states to a diversity of synchronized states, depending on species, anesthesia, and external stimulation. The average population firing rate in these states is typically low. We study analytically and numerically a network of sparsely connected excitatory and inhibitory integrate-and-fire neurons in the inhibition-dominated, low firing rate regime. For sufficiently high values of the external input, the network exhibits an asynchronous low firing frequency state (*L*). Depending on synaptic time constants, we show that two scenarios may occur when external inputs are decreased: (1) the *L* state can destabilize through a Hopf bifucation as the external input is decreased, leading to synchronized oscillations spanning d *δ* to *β* frequencies; (2) the network can reach a bistable region, between the low firing frequency network state (*L*) and a quiescent one (*Q*). Adding an adaptation current to excitatory neurons leads to spontaneous alternations between *L* and *Q* states, similar to experimental observations on UP and DOWN states alternations.

## Introduction

Electrophysiological recordings in anaesthetised, asleep and awake animals have revealed that cortical networks exhibit a diversity of dynamical states. In awake cats and monkeys, recordings seem to be compatible with an asynchronous network state in which the population firing rate is relatively constant in time, neurons receive synaptic inputs that are in average subthreshold but large fluctuations in these inputs lead to spiking^1–3^. Other studies, most prominently in rodents, have offered a different picture. In those recordings, synaptic inputs to neurons seem to be highly synchronous^4–8^. In some circumstances, recordings reveal an alternation between so-called UP states, in which neurons are depolarized compared to their resting potential, receive a large amount of excitatory and inhibitory inputs and emit spikes at a rates of a few Hz to a few tens of Hz, depending on cell type; and DOWN states, essentially quiescent states in which most neurons have their membrane potential close to the resting potential and fire very few spikes, if any^1,9–11^. Similar UP and DOWN state alternations have been observed in *in vitro* preparations^12–16^. The same networks can alternate between synchronous and asynchronous states, depending on the state of the animal (anesthetized, awake or asleep^9^), sensory stimulation^3^, and/or arousal^17^.

Most theoretical studies of cortical dynamics have focused either on asynchronous states or UP/DOWN state alternations, but have not explained how both types of dynamics could be observed in the same network and what could lead to transitions between both types of behaviors. The dominant model for asynchronous states in cortex has been the ‘balanced network’ model, in which strong excitatory and inhibitory inputs approximately cancel each other, leading to subthreshold average membrane potential, whose large fluctuations generate irregular firing at low rates^18–22^. Such a state can be shown to be stable in a wide parameter range, provided inhibition is sufficiently strong to dominate the strong positive feedback induced by recurrent excitation, and external inputs are supra-threshold. A previous analytical study of a sparsely connected network of excitatory and inhibitory leaky integrate-and-fire neurons^21^ revealed the potential presence of oscillatory instabilities of this asynchronous irregular state, both for strong external inputs (leading to fast network oscillations) and weaker external inputs (leading to slower oscillations). However, while the mechanisms of fast oscillations occurring in such networks have been studied in great detail^23–25^, slow oscillations in the low external inputs regime have been essentially unexplored.

Other studies have focused on UP and DOWN state dynamics, using purely numerical approaches^26–28^. The dominant model has been one in which strong recurrent excitation leads to a bistability between two states, an active and an inactive state^26^. In such a scenario, transitions between states are due to a slow negative feedback variable, such as an intrinsic current leading to firing rate adaptation^26^, or synaptic short-term depression^27,29^. While this scenario reproduces the basic UP/DOWN state alternation seen in experiments, it suffers from a number of shortcomings. First, these models assume a dominant role for cortical excitation, at odds with balanced network models, and with a growing body of evidence indicating the inhibition dominates the dynamics of cortical networks *in vivo* ^2,30^. Second, these models do not explain how the same network can switch between UP/DOWN state transitions and stable asynchronous irregular dynamics.

The observation of UP/DOWN state transitions suggests that the underlying network has the potential for bistability between a *low* activity state *L* (the UP state) and an essentially quiescent state *Q* (the DOWN state). Transitions between the two states then occur either due to noise, or to a slow negative feedback mechanism. Only few theoretical studies have indicated the possibility of a *L*/*Q* bistability in networks dominated by inhibition. In a randomly connected network of excitatory and inhibitory neurons^21^, showed that multiple fixed points can coexist in the inhibition dominated regime, but that in practice the potential UP state is destabilized due to an oscillatory instability. Other studies have shown, using a purely numerical approach, that in conductance-based models of spiking neurons it is in principle possible to achieve bistability between an active state at low rates and a quiescent state^31–33^. Further, it has been shown that UP/DOWN state dynamics can be achieved by adding adaptation to excitatory neurons, and coupling together two networks, representing either cortex and thalamus, or two cortical layers^32^. It remains however unclear whether such dynamics can be achieved in a single network, whose feedback is dominated by inhibition.

In the present paper, we analyze the dynamics of a sparsely connected network of LIF neurons, in a regime of strong recurrent inhibition, using both analytical and numerical tools. We identify for a broad range of parameters a region where multiple fixed points coexist, including a low activity state and a quiescent state. We show that in this region the low activity state is unstable unless excitatory synaptic time constants are significantly longer than inhibitory time constants. Finally, we show that adding firing rate adaptation leads to slow alternations between the two states, similar to UP/DOWN state alternations observed in cortical networks. Thus, this network model can serve as a minimal network model that can either settle in a stable asynchronous state (for sufficiently large external inputs) or in UP/DOWN state alternations (for intermediate values of external inputs).

## Methods

### Spiking Network Model

We consider a model network of sparsely connected excitatory and inhibitory current based LIF neurons introduced by^21^. The network is composed of *N*_*E*_ excitatory and *N*_*I*_ inhibitory neurons. Each neuron in the network receives *C* randomly chosen connections from other neurons, of which *C*_*E*_ = *cN*_*E*_ are from excitatory neurons and *C*_*I*_ = *cN*_*I*_ are from inhibitory neurons, where *c* = 0.1 is the connection probability. It also receives external inputs.

The depolarization *V*_*i*,*a*_(*t*) of neuron *i* in population *a* = *E* (excitatory) or *I* (inhibitory) obeys the equation

1 $${\tau }_{m,a}\frac{d{V}_{i,a}(t)}{dt}=-{V}_{i,a}(t)+{I}_{i,a}^{ext}(t)+{I}_{i,a}^{rec}(t)$$

where *τ*_*m*,*a*_ is the membrane time constant of neurons in population *a*, ${I}_{i,a}^{ext}(t)$ and ${I}_{i,a}^{rec}(t)$ are the external/recurrent synaptic inputs to neuron *i* in population *a*. When *V*_*i*_(*t*) reaches the firing threshold *θ*, an action potential is emitted and the depolarization is reset to the reset potential *V*_*r*_ after a refractory period *τ*_*rp*_ during which the potential is insensitive to stimulation.

The recurrent synaptic current received by a neuron *i* in population *a* = *E*,*I* are

2 $${I}_{i,a}^{rec}(t)={\tau }_{m,a}\sum _{j=1}^{{N}_{E}}{c}_{ij,aE}{J}_{aE}\sum _{k}\delta (t-{t}_{j,E}^{k}-{D}_{ij,aE})-{\tau }_{m,a}\sum _{j\mathrm{=1}}^{{N}_{I}}{c}_{ij,aI}{J}_{aI}\sum _{k}\delta (t-{t}_{j,I}^{k}-{D}_{ij,aI});$$

where the first term on the r.h.s. describes excitatory inputs, while the second term describes inhibitory inputs; *c*_*ij*,*ab*_ is the binary (0, 1) connectivity (adjacency) matrix from population *b* to population *a*, whose entries are drawn randomly subject to the constraint ∑_*j*_ *c*_*ij*,*ab*_ = *C*_*b*_; *J*_*ab*_ is the synaptic efficacy (measuring the amplitude of post-synaptic potential due to a single presynaptic spike) of synapses from a neuron in population *b* to a neuron in population *a*; ${t}_{j,a}^{k}$ is the time of the *k* − *th* spike from pre-synaptic neuron *j* in population *a*; and *D*_*ij*,*ab*_ is the transmission delay from neuron *j* in population *b* to neuron *i* in population *a*. *D*_*ij*,*ab*_ s are drawn randomly and independently from an exponential distribution with mean *D*_*ab*_ for synapses connecting population *b* to population *a*, for *a*,*b* = *E*,*I*. Instantaneous synapses with a wide distribution of delays is an analytically tractable way of describing synaptic interactions with a multiplicity of time constants : short (a few ms) time constants due to AMPA receptor activation, and longer (a few 10s of ms) time constants due to NMDA receptor activation. In Section “Spiking Network Model with firing rate adaptation” synaptic interactions are described in a more realistic fashion. Each neuron receives in addition *C*_*X*_ connections from excitatory neurons outside the network. Assuming external spike trains are uncorrelated Poisson processes with rate *ν*_*X*_ activating synapses of strength *J*_*aX*_ and using the diffusion approximation, the external synaptic current received by a neuron *i* can be written as:

3 $${I}_{i,a}^{ext}(t)={\mu }_{aX}+{\sigma }_{aX}\sqrt{{\tau }_{m,a}}{\eta }_{i}(t);$$

where *μ*_*aX*_ describes the mean input, *σ*_*aX*_ the amplitude of the fluctuations around the mean, and *η*_*i*_(*t*) are uncorrelated Gaussian white noises with 〈*η*_*i*_(*t*)〉 = 0 and 〈*η*_*i*_(*t*)*η*_*j*_(*t*′)〉 = *δ*_*ij*_ *δ*(*t* − *t*′). *μ*_*aX*_ and *σ*_*aX*_ are given by

4 $${\mu }_{aX}={C}_{X}{\nu }_{X}{J}_{aX}{\tau }_{m,a}$$

5 $${\sigma }_{aX}={J}_{aX}\sqrt{{C}_{X}{\nu }_{X}{\tau }_{m,a}};$$

The external frequency *ν*_*X*_ will be compared in the following to the frequency that is needed for a neuron to reach threshold in absence of feedback, *ν*_*θ*,*a*_ = *θ*/(*J*_*aX*_ *C*_*X*_ *τ*_*m*,*a*_).

#### Parameter choices

We choose *N*_*E*_ = 0.8*N*, *N*_*I*_ = 0.2*N* (80 % of excitatory neurons). This implies *C*_*E*_ = 4*C*_*I*_, where we set *γ* = 1/4 so that *C*_*I*_ = *γC*_*E*_. The number of connections from outside the network is taken to be equal to the number of recurrent excitatory ones, *C*_*X*_ = *C*_*E*_, and likewise, the synaptic efficacies from outside the network *J*_*aX*_ = *J*_*aE*_ for *a* = *E*,*I*. Single neuron parameters are *θ*_*E*_ = *θ*_*I*_ = 20 mV; *V*_*r*,*E*_ = *V*_*r*,*I*_ = 10 mV; *τ*_*rp*_ = 2 ms, *τ*_*m*,*E*_ = 20 ms, *τ*_*m*,*I*_ = 10 ms.

The remaining parameters are the parameters describing the coupling (the *J* and *D* two-by-two matrices); *ν*_*ext*_, the frequency of the external input and *C*_*E*_, the number of recurrent excitatory connections. To reduce further the number of parameters, we choose *D*_*EE*_ = *D*_*IE*_ and *D*_*EI*_ = *D*_*II*_. We also introduce the I/E balance parameters *g*_*E*_ = *JEI*/*JEE*, *g*_*I*_ = *JII*/*JIE*. In some cases we choose this I/E balance to be identical in both, in which case *g*_*E*_ = *gI* = *g*.

### Mean-field approach: Fixed points

We use a mean-field approach to investigate the dynamics of the network^19,21^. Using the diffusion approximation, the average firing rate *ν*_*a*0_ in population *a* = *E*,*I* in asynchronous states (i.e. states in which the instantaneous firing rates are constant in time) is given by

6 $${\nu }_{a0}={\rm{\Phi }}({\mu }_{a0},{\sigma }_{a0}),$$

where Φ is the f-I curve of the LIF neuron in the presence of white noise^34,35^,

7 $${\rm{\Phi }}(\mu ,\sigma )={[{\tau }_{rp}+\sqrt{\pi }{\tau }_{m}{\int }_{\frac{{V}_{r}-\mu }{\sigma }}^{\frac{\theta -\mu }{\sigma }}dx{e}^{{x}^{2}}[1+erf(x)]]}^{-1},$$

where erf is the standard error function, while *μ*_*a*0_ and *σ*_*a*0_ represent the mean inputs and magnitude of the temporal fluctuations in inputs,

8 $${\mu }_{a0}={\tau }_{m,a}{C}_{E}{J}_{aE}({\nu }_{X}+{\nu }_{E0}-\gamma {g}_{a}{\nu }_{I0})$$

9 $${\sigma }_{a0}^{2}={\tau }_{m,a}{C}_{E}{J}_{aE}^{2}({\nu }_{X}+{\nu }_{E0}+\gamma {g}_{a}^{2}{\nu }_{I0})$$

The equations are solved numerically by introducing sets of ODEs whose fixed points are given by Eqs ((6)–(9)) and integrating these ODEs until they converge to a fixed point (see Appendix A for more details). Comparisons between mean-field analysis and numerical simulations are shown in Appendix B.

### Mean-field approach: Stability analysis of fixed points

To study the stability of fixed points, we performed a linear stability analysis of the stationary solutions of the Fokker-Planck equations describing the probability distribution of membrane potentials in both populations, as well as the associated instantaneous firing rates^21,36–38^. Details of this stability analysis are presented in Appendix C.

## Results

### Existence of multiple fixed points in the inhibition dominated region

We started by exploring parameter regions in which multiple fixed points of network activity exist, using mean-field equations described in Methods. For a given set of parameters, we solve numerically mean-field equations ((6)–(9)) to compute the firing rates in the asynchronous states of the network. We choose to present the results in the *g* − *ν*_*X*_/*ν*_*θ*,*E*_ plane, for fixed values of *C*_*E*_ and *J* ^21^.

**Figure 1:**
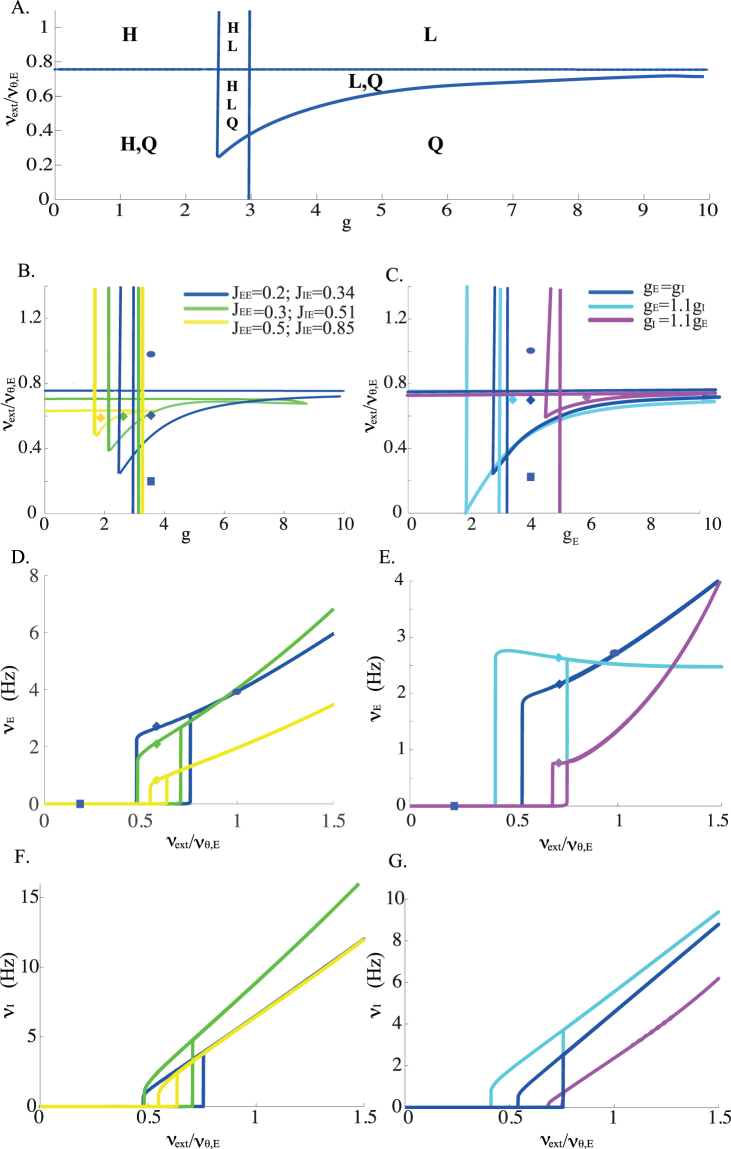
(**A**) Regions of existence of the different types of stationary solutions in the external input/ inhibition strength plane. Bifurcation diagram of stationary solutions as a function of the external input and inhibition strength. Blue lines: saddle-node bifurcations. These saddle-node bifurcation lines define 7 regions, in which different combinations of states can exist. Potentially stable fixed points in each of the regions are: *H*, high firing rate; *L*, low firing rate; *Q*, quiescent state; *H*,*L*, *L*,*Q* and *H*,*Q* denote the regions in which two types of solutions coexist; Finally, in *H*,*L*,*Q* all three types of states coexist. Parameters: *C*_*E*_ = 1000, *J*_*EE*_ = 0.2 mV, *J*_*IE*_ = 0.34 mV. *g*_*E*_ = *g*_*I*_ = *g*. (**B**) Bifurcation diagrams for increasing coupling strengths (all connections are multiplied by the same factor). (**C**) Bifurcation diagrams for different I/E balance parameters *g*_*E*_/*g*_*I*_. (**D**) Average firing frequency of the excitatory population as function of the external input, for coupling strengths indicated in B. Symbols indicate the value of *g* chosen for each curve. (**E**) Average firing frequency of the excitatory population as function of the external input, for *g*_*E*_/*g*_*I*_ indicated in C. Vertical lines indicate saddle-node bifurcations that delimit the region of coexistence *L*,*Q*. (**F**) Same as in D. but for the inhibitory population. (**G**) Same as in E. but for the inhibitory population.

We show in Fig. 1A the ‘phase diagram’ in the *g* − *ν*_*X*_/*ν*_*θ*,*E*_ plane, for *C*_*E*_ = 1000, *J*_*EE*_ = 0.2 mV, *J*_*IE*_ = 0.34 mV. *g*_*E*_ = *g*_*I*_ = *g*. This phase diagram shows seven qualitatively different regions, in which one, three or five fixed points exist, out of which one, two or three are potentially stable (see below). The potentially stable fixed points are: *H*, a high firing state (close to saturation), which exists in the weak inhibition region, up to a critical value of *g* ($g\sim 3$ in Fig. 1A); *L*, a low but non-zero firing state, which exists in the strong inhibition region ($g\sim 2.5$ in Fig. 1A), and above a critical value of external inputs, that depend on *g*; *Q*, a quiescent state, in which the firing rate is extremely small, that exists when the external inputs are sufficiently far from threshold (*ν*_*X*_ < 0.78*ν*_*thr*_ in Fig. 1A). All these fixed points appear/disappear on saddle-node bifurcation lines (indicated by blue lines), together with the associated unstable fixed points. There are a total of 4 regions in which multiple fixed points coexist. All possible combinations are possible.

In the following, we focus on the inhibition-dominated regime, in which the *H* state does not exist. In this regime, there exists a region with three fixed points, out of which two are potentially stable: the *Q* and the *L* fixed points. We next study how the size of the *L*/*Q* coexistence region depends on the parameters characterizing the strength of the coupling. First, this region exists provided *J*_*IE*_ *τ*_*I*_ is sufficiently below *J*_*EE*_ *τ*_*E*_^21^. When this condition is fulfilled, the mean external inputs to excitatory neurons is stronger than the mean external inputs to inhibitory neurons, which creates a window of opportunity for a coexistence region between the *Q* and *L* states. If *J*_*IE*_ becomes stronger, then the coexistence region is destroyed since inhibition becomes too powerful to sustain it. Decreasing *J*_*EE*_ together with *J*_*IE*_ so as to keep their ratio constant, increases the size of the *L*/*Q* region (Fig. 1B). This effect can again be understood by considering that *J*_*EE*_ *τ*_*mE*_ *J*_*IE*_ *τ*_*mI*_. In this scenario, the coexistence of *L* and *Q* is favored by the fact that the mean external inputs to E neurons are stronger than the mean external inputs to I neurons (by a factor proportional to the ratio *J*_*EE*_ *τ*_*mE*_/*J*_*IE*_ *τ*_*mI*_). Decreasing all synaptic coupling strengths maintains this ratio constant, but decreases the size of the fluctuations in the inputs, weakening inhibition close to the bifurcation leading to the appearance of the *L* state, thereby moving this bifurcation down (i.e. *L* appears for smaller external input values).

Varying *g*_*I*_, i.e. the strength of inhibition on inhibitory neurons, with respect to *g*_*E*_, i.e. the strength of inhibition on excitatory neurons, has a different effect (Fig. 1C). It does not affect significantly the bifurcation leading to the appearance of the *L* state at large values of *g*_*E*_, but changes the location of the quasi-vertical saddle-node bifurcation. Increasing *g*_*I*_ weakens inhibition, so the *L* state destabilizes to the *H* state at higher values of *g*_*E*_. Figure 1D–G) shows that the firing rates of both E and I populations are in the order of a few spikes per second.

### Stability of the *L* and *Q* stationary solutions

The next step is to investigate the stability of the *L* and *Q* solutions. We perform a linear stability analysis of the corresponding stationary solutions of the Fokker-Plank equation, which describes the evolution of the probability distribution of a neuron depolarization^21^ (Appendix C). The analysis boils down to solving an eigenvalue equation. The sign of the real part of the eigenvalues *λ* establishes whether perturbations are amplified and an instability arises (*Re*(*λ*) 0), or, conversely, whether perturbations decay (*Re*(*λ*) < 0). The stationary solution is therefore stable provided all eigenvalues have negative real parts. The points at which the eigenvalue with the largest real part satisfies *Re*(*λ*) = 0 indicate a bifurcation, which can be either a saddle-node or a Hopf bifurcation, depending on whether the eigenvalue is real or imaginary.

**Figure 2:**
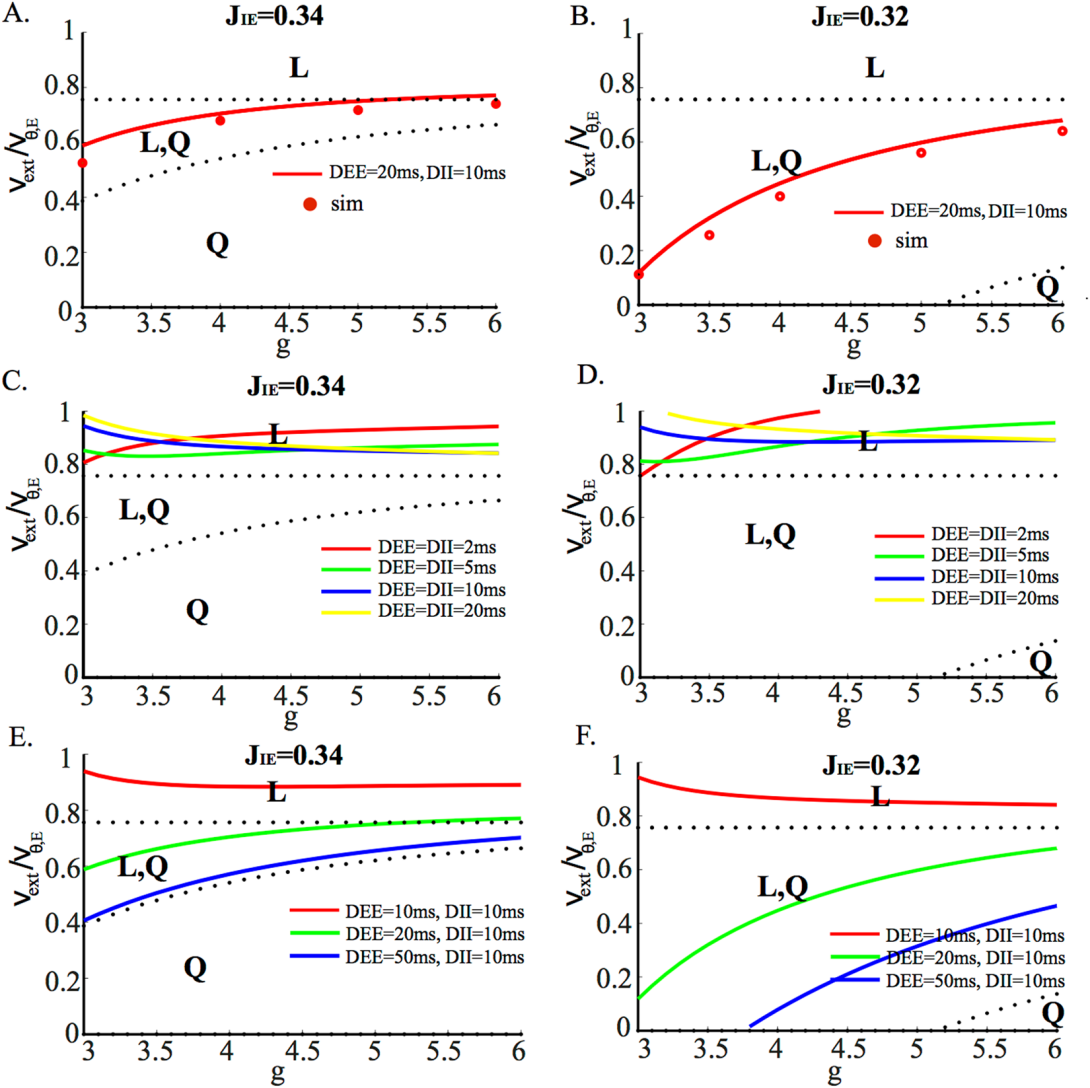
Stability region of the *L* solution (colored lines: Hopf bifurcation curves; dashed lines: saddle-node bifurcation curves) as a function of the external input and inhibition strength for different values of the average synaptic delays and for different *E* to *I* synaptic efficacies, labelled on top of each plot. (**A,B**) Red lines: Hopf bifurcation line for *D*_*E*_ = 20 ms, *D*_*I*_ = 10 ms. Red circles: Simulations (see text). (**C,D**) Hopf bifurcation lines for different values of the delay, which is here identical in excitatory and inhibitory synapses. (**E,F**) Hopf bifurcation lines for different ratios between excitatory and inhibitory delays. The stability of the *L* state increases significantly when the ratio *D*_*EE*_/*D*_*II*_ increases. Other parameters: *C*_*E*_ = 1000, *J*_*EE*_ = 0.2 mV.

**Figure 3:**
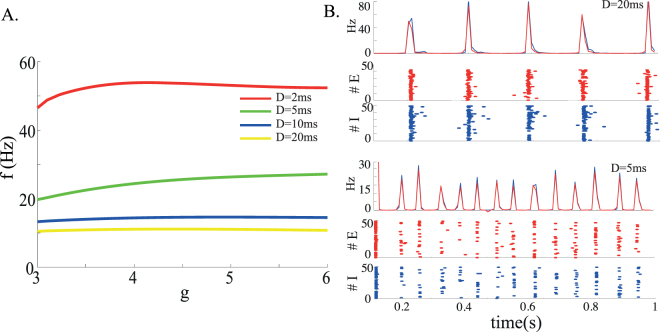
(**A**) Frequency of the network global oscillations at the onset of the Hopf bifurcation as a function of the inhibition strength *g* for a network in which excitatory and inhibitory neurons have different characteristics. Longer synaptic delays give rise to slower oscillations. In this regime, the average synaptic delays are the same for excitatory and inhibitory synapses. (**B**) Network simulations for two different average delays. Top panels: *D* = 20 ms, *ν*_*X*_/*ν*_*θ*,*E*_ = 0.78, *g* = 4; bottom panels: *D* = 5 ms, *ν*_*X*_/*ν*_*θ*,*E*_ = 0.825, *g* = 5. For both parameter sets, we show the average instantaneous firing rates of excitatory (red) and inhibitory (blue) neurons, and raster plots are plotted of 50 excitatory and 50 inhibitory neurons. Other parameters: *C*_*E*_ = 1000, *J*_*EE*_ = 0.2 mV, *J*_*EI*_ = 034 mV.

The stability analysis shows that as the external inputs are decreased from the supra-threshold region, the low rate asynchronous state destabilizes through a Hopf bifurcation (see Fig. 2A,B for two different parameter sets). However, the location of this Hopf bifurcation depends strongly on the average synaptic delays. This oscillatory instability is due to excitatory-inhibitory interactions. When the average synaptic delays are equal for E and I synapses, the *L* state tends to destabilise outside the coexistence region *L*/*Q* (Fig. 2C,D) which becomes therefore unreachable. In this regime the network exhibits pronounced oscillations whose frequency vary between a few Hz and $\sim 50$ Hz depending on average delays and *g*, as shown in Fig. 3A. Simulations confirm the presence of these oscillations below the instability line, with a frequency that decreases as the external input is decreased (Fig. 3B). In the examples shown in Fig. 3B, the population frequency is close to 5 Hz for *D* = 20 ms (top), and about 14 Hz for *D* = 5 ms (bottom). In the *D* = 20 ms case, neurons have a firing rate that is close to the population frequency, while in the *D* = 5 ms case, the firing frequencies of both E and I populations are much lower than the global oscillation frequency (around 3/s).

When the average E synaptic delays are larger than the I delays, the stability region of the *L* state expands considerably (compare panels C, D of Fig. 2 with panels E, F). Note that it is not unreasonable to expect that average E synaptic time constants are longer than I time constants, because a large fraction of the charge transmitted by EPSCs in cortex is mediated by NMDA receptors^39^. Moreover, decreasing the strength of inhibition, through a decrease of the synaptic efficacy *J*_*IE*_, not only makes the coexistence region appear for smaller values of the external input and survive for larger *g* values, but also enlarges the stability region of the *L* solution. In fact, in Fig. 2F, the *L* state survives up to the point where no external inputs are present. When the Hopf bifurcation line lies within the coexistence region (as in the case of the red line in Fig. 2A,B), numerical simulations show that the amplitude of the oscillation increases rapidly until the network falls in the quiescent state. The value of the external inputs at which this happens is very close to the analytically computed Hopf bifurcation lines, as shown by the comparison between solid red line and red circles in Fig. 2A,B). Finally, note that the *Q* solution is stable in all the coexistence region *L*/*Q* and only disappears through the saddle node bifurcation by coalescing with the unstable fixed point separating the *L* and *Q* states.

### Transitions between UP and DOWN states in the bistable region

To reproduce the observed phenomenology of UP and DOWN states, we now focus on the region in which the mean field analysis reveals a coexistence between the *L* (UP) state and the *Q* (DOWN) state. UP and DOWN state transitions could then in principle arise in two possible ways: (1) from stochastic jumps between these two fixed points, due to finite-size effects; (2) from slow oscillations between *L* and *Q* states, caused by an additional variable that provides negative feedback with a slow time constant. The latter scenario will be studied in the following sections when considering the effects of firing rate adaptation.

**Figure 4:**
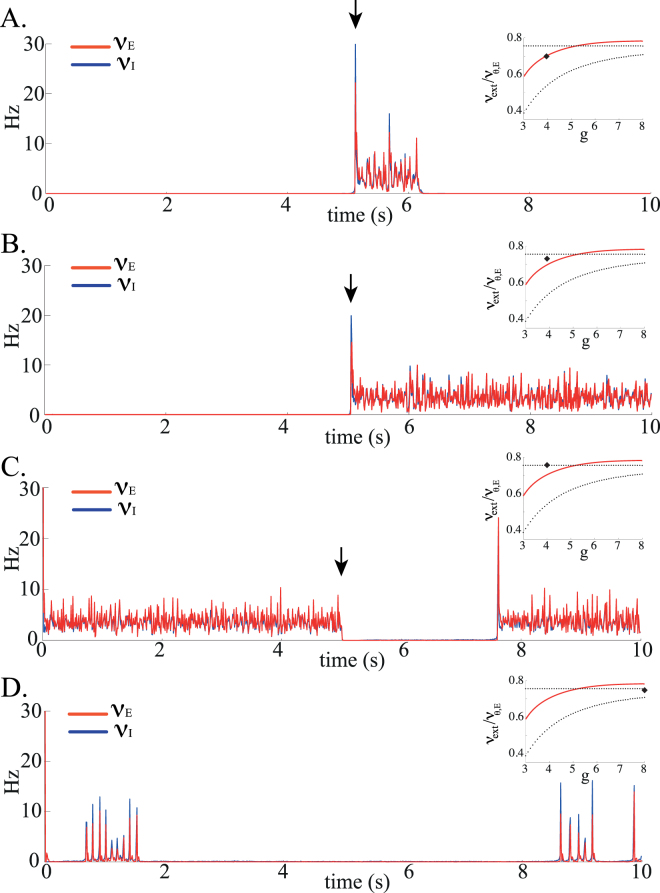
Transitions between UP and DOWN states in network simulations. (**A**) The external input is set close to the point at which the *L* state destabilises, as indicated by the black symbol in the inset (a zoomed-in version of Fig. 2A), and sufficiently far from the point at which *Q* destabilises. Network is initialized in the DOWN state. At *t* = 5 s, a pulse triggers a DOWN to UP transition (indicated by a black arrow). It is followed by a spontaneous transition from UP to DOWN after about 1 s. (**B**) The external input is set in the middle of the coexistence region *L*,*Q*. Consequently, both states have very long life times. Here the network is initialized in the DOWN state, and a pulse again triggers a DOWN to UP transition at *t* = 5 s. (**C**) Close to the point at which the *Q* state destabilises, DOWN states are much shorter than UP states. Here, the network is initialized in the UP state, and a pulse triggers an UP to DOWN transition at *t* = 5 s. (**D**) In a regime in which the points of destabilisation of *L* and *Q* are very close, which implies fine tuning of both external input and inhibition strength, UP and DOWN states have comparable durations of the order of few seconds. Parameters: *C*_*E*_ = 1000, *J*_*EE*_ = 0.2 mV, *J*_*EI*_ = 0.34 mV, *D*_*EE*_ = 20 ms, *D*_*II*_ = 10 ms. Parameters of the external stimulation (pulse): Increase (**A,B**) or decrease (**C**) of the external input to all neurons by $\sim \mathrm{15 \% }$, during 250 ms.

Network simulations show that sufficiently far from the bifurcation points, both states are stable over long time scales (tens of seconds or longer, see Fig. 4B in which an external stimulation is needed to cause a transition from the DOWN to the UP state). To obtain shorter lifetimes of these states, one needs to fine tune the external inputs so that they are close to the boundary of the stability region of the corresponding state, in order to cause noise-induced, spontaneous transitions from one state to the other. For instance, in Fig. 4A, the external inputs are very close to the Hopf bifurcation leading to destabilization of the asynchronous UP state. In this case, UP states have short lifetimes (of order 1s), but DOWN states have very long life times - in A, an external input is needed to provoke a transition from the DOWN to the UP state at *t* = 5 s. Figure 4C shows the opposite scenario of an external input close to the saddle-node bifurcation where the DOWN state disappears. In this case, DOWN states have short life times (of order 1s) but UP states are much more stable (again, external stimulation is needed to interrupt temporarily the UP state). Hence, the phenomenology of UP and DOWN states *in vitro* (very long DOWN states, ≈1 s long UP states) can be reproduced only when the external inputs are fined tuned to be close to the bifurcation where the *L* state destabilizes (Fig. 4A), while the *in vivo* phenomenology (both DOWN and UP states lasting approximately 1 s) can only be observed if two parameters are simultaneously fine tuned: a parameter controlling the size of the coexistence region of UP and DOWN states, and the external inputs (Fig. 4D).

#### Spiking Network Model with firing rate adaptation

A well established mechanism to control the time scale of UP states is firing rate adaptation^26,32^. Firing rate adaptation is prominent in pyramidal cells, while it is much weaker in fast-spiking interneurons^16,40^. We therefore investigated a network in which excitatory neurons (but not inhibitory neurons) are endowed with an adaptation current. The equation for the membrane potential of excitatory neurons becomes:

10 $${\tau }_{m,E}\,\frac{d{V}_{i,E}(t)}{dt}=-{V}_{i,E}(t)+{I}_{i,E}(t)-{A}_{i,E}(t)$$

11 $${\tau }_{A}\,\frac{d{A}_{i,E}}{dt}=-{A}_{i,E}$$

When the potential *V*_*i*,*E*_ reaches the firing threshold *θ*, a spike is emitted, the adaptation current *A*_*i*,*E*_ is increased by *β*/*τ*_*A*_ and *V*_*i*,*E*_ is reset to the resting potential *V*_*R*_. Thus, *β* controls the strength of adaptation (it corresponds to the integral of the *A* variable following a single spike), while *τ*_*A*_ is the decay time constant of the adaptation current.

**Figure 5:**
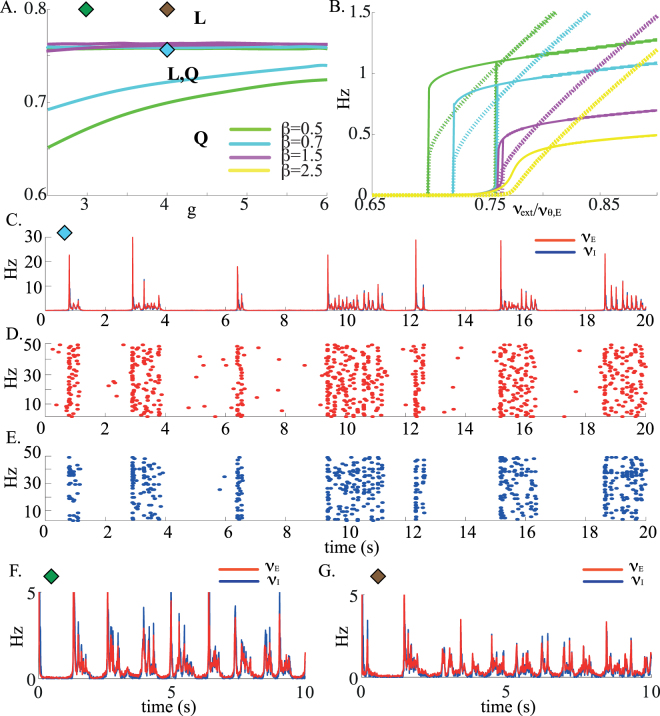
Networks of LIF neurons with firing rate adaptation. (**A**) Mean-field: Coexistence regions between *Q* and *L* states, with increasing values of the strength of adaptation *β*. The coexistence region progressively shrinks and disappears at $\beta \,\sim$1.5 mV.s. Diamonds indicates the location of the simulations shown in C, D, E, F and G. Parameters: *C*_*E*_ = 1000, *J*_*EE*_ = 0.2 mV, *J*_*EI*_ = 0.34 mV. (**B**) Bifurcation diagrams (firing rates of E and I populations vs external input) for increasing values of the strength of adaptation. Solid lines: average excitatory firing rate; Dashed lines, average inhibitory firing rate. Note that the coexistence region is no longer present for *β* = 2.5 mV.s. (**C–E**) Network simulations for *g* = 4, *ν*_*X*_ = 0.76*ν*_*θ*,*E*_, *β* = 0.7 mV.s, *τ*_*A*_ = 200 ms, *D*_*EE*_ = 20 ms, *D*_*II*_ = 10 ms. (**C**) Average *E* and *I* firing frequency as a function of time. The population firing frequency is averaged over 10 ms time bins. Note UP and DOWN states with irregular durations of order 1 s, and instantaneous fluctuations of E and I neurons in UP states that closely track each other. (**D**) Raster of 50 excitatory neurons (**E**) Raster of 50 inhibitory neurons. (**F**) Network simulations for *g* = 3, *β* = 5 mV.s, *τ*_*A*_ = 500 ms, *ν*_*X*_ = 0.8*ν*_*θ*,*E*_. (**G**) Network simulations for *g* = 4, *β* = 5 mV.s, *τ*_*A*_ = 600 ms, *ν*_*X*_ = 0.8*ν*_*θ*,*E*_.

To study the effect of adaptation on the *L*/*Q* bistable range, we first modified the mean-field equations to account for the adaptation current, using an adiabatic approximation that replaces adaptation currents by their mean^41^ (Appendix D). Figure 5A,B show that the stronger the adaptation, the smaller the bistable range, as one would expect, until the bistable range disappears for $\beta \sim$1.5 mVs. Furthermore, the firing rates of both excitatory and inhibitory populations also gradually decrease as a function of the adaptation variable.

We first focused on a parameter region such that the bistable region between *L* and *Q* states is still present, but reduced in size due to adaptation (*β* = 0.7 mV.s, see blue diamond in Fig. 5A; compare the extent of the *L*/*Q* region with Fig. 2A). In this regime, *L* and *Q* states are indeed stable on short time scales, but they destabilize on longer time scales due to noise-induced transitions. As a result, both UP and DOWN states have highly variable durations (Fig. 5C,D,E - see Appendix G for details on how durations are computed). In this regime, the external input still needs to be finely tuned in order to observe realistic lifetimes of UP and DOWN states, but only a single parameter needs to be fine tuned in order to obtain UP/DOWN state durations of the order of 1 s. In practice, the external inputs needs to be close to the bifurcation leading to the disappearance of the *Q* state. The presence of adaptation considerably shortens the duration of UP states, that are also of the order of 1s.

We next investigated a scenario in which the bistable region has completely disappeared due to adaptation (*β* = 5 mV.s, see green and brown diamonds in Fig. 5A). In this regime, adaptation generates slow oscillations between UP and DOWN states, whose time scales is controlled by the adaptation time constant (see Fig. 5F,G). This scenario is similar to the one discussed in^42^ and^26^. UP and DOWN states in this scenario have much less variable durations - CV of durations of UP/DOWN states are in the range 0.4–0.6/0.1–0.2, respectively, approximately three times smaller than the values observed in Fig. 5C.

**Figure 6:**
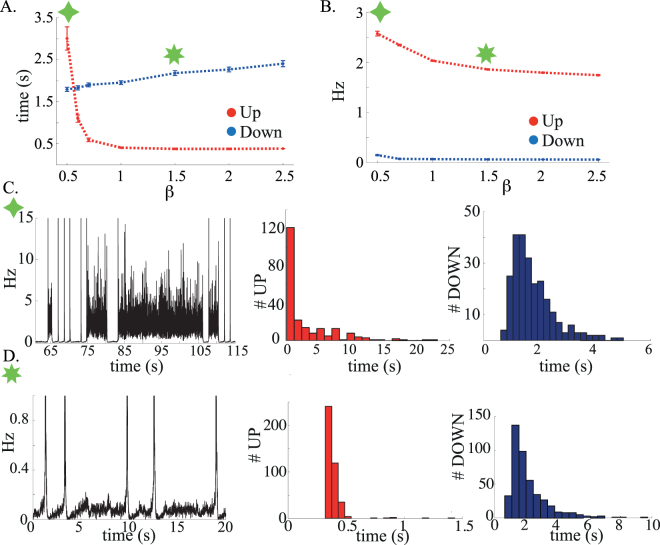
(**A**) Average UP and DOWN states duration as a function of the adaptation strength. (**B**) Average excitatory firing frequency during UP and DOWN states as a function of the adaptation strength. Each point in A. and B. is the average duration or firing frequency of a sample of UP and DOWN states collected over 1500s of network simulation; error bars are S.E.M. Up and DOWN states are detected through the crossing points of a fast and a slow exponential moving average. (**C**) Exemplary sequence of UP and DOWN states versus time for the adaptation value labelled by the green diamond and corresponding histograms of UP and DOWN states durations. (**D**) Same as in C. but for a different adaptation strength, labelled by the green star. Parameters: *J*_*EE*_ = 0.2 mV, *J*_*EI*_ = 0.34 mV, *g* = 3, *τ*_*A*_ = 0.2 s, *ν*_*X*_ = 0.76*ν*_*θ*,*E*_, *D*_*EE*_ = 20 ms, *D*_*II*_ = 10 ms.

Figure 6A shows the average time duration of UP and DOWN states in the noise-induced transition regime, as a function of the adaptation strength *β*, computed from network simulations. The smaller *β*, the longer is the *UP* state, which terminates due to the variance of the synaptic input. This stochasticity of the UP/DOWN transitions is reflected in the wide distributions of time durations shown in Fig. 6C. For larger *β* values (Fig. 6D), the *L* state is quickly pulled towards *Q* by the adaptation, whose contribution is now at least as large as the synaptic input fluctuations. In this case, the mean duration of the UP state is much shorter and its distribution narrower than in the previous case. On the other hand, DOWN states duration is essentially unaffected by the adaptation strength, since firing rates are so low in the *Q* state that the adaptation current is essentially negligible. Note that contrary to UP states, DOWN state durations cannot be smaller than a few hundreds ms. This is due to the fact that it takes time for the adaptation current to decay to a very small value after transitions from *L* to *Q*, making it harder for the network to make a transition back to the UP state during that interval. The adaptation current lowers the average population firing frequencies of the network in the UP state, but has very little effect on the firing frequencies of the DOWN state, which are close to zero for any *β* value (Fig. 6B).

**Figure 7:**
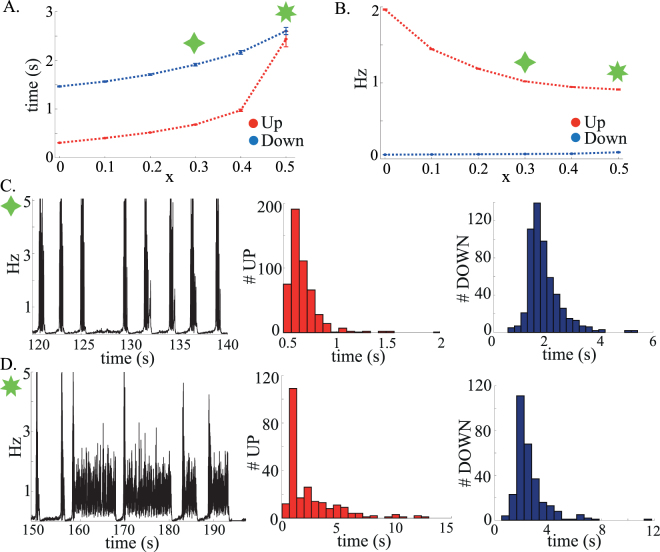
(**A**) Average UP and DOWN states duration as a function of the fraction of charge mediated by NMDA receptors. (**B**) Average excitatory firing frequency during UP and DOWN states as a function of the fraction of slow recurrent inputs. (**C,D**) Same as in Fig. 6. Parameters: *J*_*EE*_ = 0.2 mV, *J*_*EI*_ = 0.34 mV, *g* = 4, *τ*_*A*_ = 0.3*s*, *ν*_*X*_ = 0.76*ν*_*θ*,*E*_, *τ*_*AM*_ = *τG* = 5 ms, *τ*_*N*_ = 50 ms.

We also simulated networks with more realistic synaptic currents (see Appendix E for details). Excitatory currents were mediated by a combination of slow (NMDA-like) and fast (AMPA-like) synaptic currents, while inhibitory currents were mediated by fast, GABA_*A*_-like, synaptic currents. We found that this more realistic network model exhibited very similar dynamics as the network with instantaneous PSCs. The presence of slow NMDA-like synaptic excitation played the same role as the longer average excitatory delays in the network with instantaneous PSCs in stabilizing the UP state well into the coexistence region. Figure 7A shows that the duration of both UP and DOWN states increase as a function of the fraction of charge *x*_*E*_ mediated by the slow NMDA receptors. This is due to the fact that NMDA stabilizes both states, by reducing the magnitude of fluctuations in the recurrent inputs. The distributions of both states (Fig. 7C,D) show again an exponential tail, consistent with stochastic transitions.

**Figure 8:**
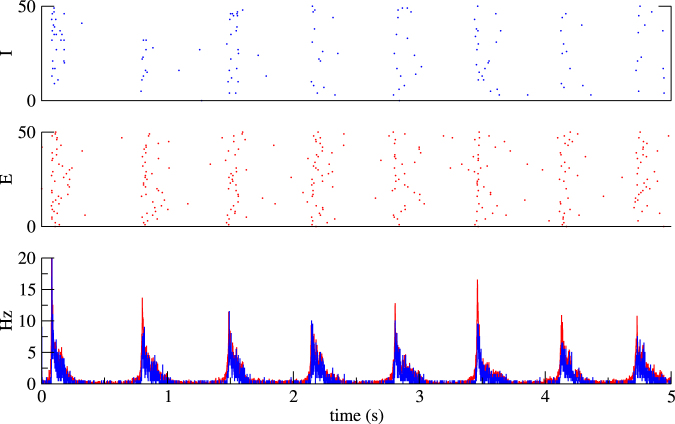
UP and DOWN states in networks with conductance-based synapses (see Appendix F for details). Parameters: *τ*_*E*_ = 10 ms, *τ*_*I*_ = 5 ms, *ν*_*X*_ = 0.8 *ν*_*thr*_, *γ* = 0.5. Other parameters as in Fig. 6, converted into conductances as described in Appendix F.

Finally, we checked that our results are qualitatively valid in networks with conductance-based synapses (see Appendix F for details). In particular, bistability between low activity states and quiescent states occur in large regions of parameters^43,44^. Adding an adaptation current to excitatory neurons readily converts this bistability into an alternation between UP and DOWN states, as shown in Fig. 8, which shows a slow oscillation with a frequency of about 1.6 Hz.

## Discussion

We have explored the dynamics of a sparsely connected network of excitatory and inhibitory spiking neurons, in a strong recurrent inhibition, low firing rate regime. Our network is the simplest analytically tractable spiking network model which can exhibit both stable asynchronous irregular dynamics and UP/DOWN state alternations, and switch between the two regimes by changing the external inputs. The size of this bistable range is controlled by all parameters describing the connectivity between neurons, such as numbers and strengths of excitatory and inhibitory connections. We investigated the stability of the *L* state as the external inputs are decreased, and show that it generically destabilizes through a Hopf bifurcation, leading to oscillations whose frequency is determined by synaptic time constants. When synaptic time constants are short, the Hopf bifurcation appears for values of the external input that are above the *L*/*Q* coexistence range, preventing any bistability. On the other hand, longer synaptic time constants lead to a stabilization of the *L* state deep into the coexistence region. Oscillations appearing beyond the Hopf bifurcation outside the coexistence range have frequencies that range from a few Hz to a few tens of Hz.

In the *L*/*Q* bistable regime, we show that noise-induced transitions can occur between the two states, giving rise to UP and DOWN state alternations. The lifetime of these states are however extremely long, unless parameters are chosen to be close to the boundaries of the bistable range. Finally, we investigated the effect of firing rate adaptation on excitatory neurons. We showed that weak to moderate firing rate adaptation reduces the size of the bistable range, alleviating to some extent the need to fine tuning to obtain realistic lifetimes of UP and DOWN states. For strong adaptation, bistability is destroyed, and is replaced by an oscillatory regime in which the network alternates rhythmically between UP and DOWN states.

In our model, different experimentally observed regimes can be reproduced by varying a single parameter, the frequency of external inputs. *In vivo*, external inputs represent inputs coming to the network from sub-cortical structures, nearby cortical networks through lateral connections, and inputs from other cortical areas. *In vitro*, external inputs represent spontaneous neurotransmitter release^45^, or spontaneous activity in subsets of neurons. For sufficiently high external inputs, the network exhibits a stable asynchronous state while, for lower external inputs, it switches to a synchronized state due to a variety of potential mechanisms (i) slow stochastic UP/DOWN state alternations; (ii) UP/DOWN alternations due to firing rate adaptation; or (iii) oscillations due to E/I feedback loop, whose frequency can range from a few Hz to a few tens of Hz.

In the first scenario, transitions between stable states are due to fluctuations in the network spiking activity due to finite size effects. In this case, fine tuning is needed to achieve UP and DOWN states lifetimes that are consistent with data. However, the fine tuning problem would be strongly alleviated in the presence of slow global fluctuations in external inputs or in the cellular excitability. The time scales of this slow variable would then determine, to a large extent, the time scales of UP and DOWN state transitions.

In the second scenario, the asynchronous state destabilizes due to the slow negative feedback induced by firing rate adaptation^42,46–48^. In both the noise-induced and the adaptation-induced regimes, our network can reproduce the diversity of phenomenologies of durations of UP/DOWN states seen in different experiments: with weak external inputs, UP states are much shorter than DOWN states, similar to what is seen *in vitro* ^12^. *In vivo*, the opposite often occurs^49–54^. Transitions between UP and DOWN states often seem irregular and stochastic, as in our model^50,52,55,56^. These stochastic transitions between UP and DOWN states could explain to a large extent noise correlations in cortical circuits^54^.

In the third scenario, the oscillatory instability is due to the E/I interactions, similar to the classic E-I instability that occurs in many E-I networks, including firing rate models^57^. In this case, the asynchronous state destabilizes outside the bistable region, i.e. for higher values of the external input, and the network generate oscillations with a frequency that depends on synaptic delays.

The dependence of the dynamics on external inputs is consistent with experimental observations, where there is a general trend of observing synchronous dynamics in asleep and anesthetized preparations, while the network tends to be more asynchronous in awake animals^1,9^. Furthermore, recordings in awake monkeys are consistent with transitions from synchronized activity in the absence of sensory stimulation, to asynchronous activity in the presence of such a stimulation^3^. Similar observations have been made as a function of arousal, as measured by pupil diameter^17^. In all scenarios, the frequency of UP/DOWN state alternations increases with external inputs. This is again consistent with experimental observations showing slow frequencies *in vitro* (1 Hz or less), while synchronized activity *in vivo* is often characterized by faster frequencies in the 1–5 Hz range^3,7^.

The inhibition dominated regime we have studied here is consistent with a large body of *in vitro* ^16^, as well as *in vivo* data^2,30,58–60^. However, other experiments have found results consistent with a dominance of weak and sparse excitatory inputs^5^. Interestingly, these results can also be reconciled with our model, which often produces I rates that are lower than E rates for low values of external inputs (see Fig. 1). In rodent cortex, whether networks are inhibition dominated (and in particular inhibition stabilized) is still an open question (see e.g. ref.^61^).

Our work investigates the dynamics of standard LIF network of E and I neurons in the low rate regime in greater detail than in previous studies of the same network^21^. We show that the discrepancies between numerical and analytical results are due to deviations from the diffusion approximation, and disappear when one uses a transfer function Φ derived from a shot-noise process.

Our scenario for the emergence of UP/DOWN state transitions is distinct from most previous studies, which relied on an excitation dominated network with a slow negative feedback leading to slow UP and DOWN states alternations^26,27,62,63^. In these models rhythmic alternations are elicited in the absence of noise. We propose a different scenario of an inhibitory-dominated network in which bistability can arise when the mean external inputs are stronger in excitatory neurons than in inhibitory neurons. This scenario is similar to the one proposed recently in simpler firing rate models^64,65^. We showed that in LIF networks, bistability can be achieved provided excitatory synaptic time constants are slower than inhibitory time constants.

Consistently with^33^, we find bistability of LIF networks for sub-threshold values of external inputs, confirming that bistability between quiescent and active states is not an exclusive property of conductance-based networks^31,32^. Our model and analysis bear similarities with a recently published study which investigated the interplay between bistability and oscillations in both a simple excitatory-inhibitory firing rate model, and a network of LIF neurons, close to a Takens-Bogdanov bifurcation^66^. One difference with the Roxin and Compte contribution is that they considered a fully connected network with 1/*N* coupling in the large *N* limit, while we analyzed a randomly connected network with finite coupling using the diffusion approximation. Consequently, our analysis (similarly to^21^) takes into account the fluctuations in the recurrent inputs, that vanish in the fully connected network with 1/*N* coupling. However, while variance dynamics can in some circumstances have a strong impact on the dynamics of the network^67^, it has a relatively minor impact on the location of both saddle-node and Hopf bifurcations for the parameters that were considered here.

The model makes several testable predictions. First, we predict that transitions from sleep (UP and DOWN oscillations) to wakefulness (asynchronous irregular activity) is controlled to a large extent by the magnitude of external inputs. Second, the average population firing frequency in the asynchronous state is higher than the firing frequency during an UP state. This holds true for both E and I populations, although the increase in firing rate is larger for I than for E (Fig. 1D to G).

Our model is both realistic enough to generate both asynchronous states at low rates and UP/DOWN state alternations, but also simple enough to allow us to study it analytically. It should be possible to generalize it in a number of directions. For instance, it would be interesting to use conductance-based, rather than current-based, synaptic inputs^68^. It would also be of interest to perform the linear stability analysis of the master equation describing the dynamics of the distribution of membrane potentials in the presence of shot-noise inputs^69^. Another promising extension would be to generalize the mean-field analysis to a network composed of multiple layers and/or multiple interneuron types^70^, in order to clarify the specific roles of each layer and/or cell type in the generation of UP/DOWN state alternations. Finally, it would be worthwhile to be able to capture analytically the statistics of the durations of both UP and DOWN states, similarly to what has been done in simpler models^71^.

### Data availability

All the codes used for generating the figures in this paper are available upon request.

## Electronic supplementary material

Bistability and up/down state alternations in inhibition-dominated randomly connected networks of LIF neurons
