# Supplementary material for: Bistability and up/down state alternations in inhibition-dominated randomly connected networks of LIF neurons
Source: Sci Rep. 2017 Sep 20;7:11916. doi: 10.1038/s41598-017-12033-y (PMC5607291; doi:10.1038/s41598-017-12033-y)
Supplement: Supplementary file 1 — Bistability and up/down state alternations in inhibition-dominated randomly connected networks of LIF neurons [file 41598_2017_12033_MOESM1_ESM.pdf]

Bistability and up/down state alternations in inhibition-dominated randomly connected networks of LIF neurons

Elisa M. Tartaglia[1,2,3*] and Nicolas Brunel[2,3,4,5]

[1]UPMC Univ Paris 06, UMRS 968, Institut de la Vision, Paris, France.
[2]Department of Neurobiology, The University of Chicago, Chicago, Illinois, USA.
[3]Department of Statistics, The University of Chicago, Chicago, Illinois, USA.
[4]Department of Neurobiology, Duke University, Durham, NC, USA.
[5]Department of Physics, Duke University, Durham, NC, USA.
[*]elisanitamaria@gmail.com

July 27, 2017

Appendix

A. Solving numerically mean-field equations

To solve numerically the mean-field equations, we introduce ODEs whose fixed points satisfy Eqs. (6-9),

$$\tau_E \dot{\nu}_E(t) = -\nu_E(t) + \Phi\left(\mu_E(t), \sigma_E(t)\right) \quad (1)$$
$$\tau_I \dot{\nu}_I(t) = -\nu_I(t) + \Phi\left(\mu_I(t), \sigma_I(t)\right) \quad (2)$$

where μ_as and σ_as are functions of $\nu_a(t)$s through Eqs. (8-9). To get convergence to fixed points, we can use $\tau_I \ll \tau_E$. Typically we use $\tau_I = 0.1\tau_E$.

We emphasize that these equations do not describe the real dynamics of the system (which are given by the full system of Fokker-Planck equations, Eq. (12); it is only a numerically convenient tool to find the fixed points of the mean-field equations.

B. Agreement between spiking simulations and mean field theory

While all the features described by the mean-field approach are reproduced qualitatively by numerical simulations, there are significant quantitative discrepancies between both, as

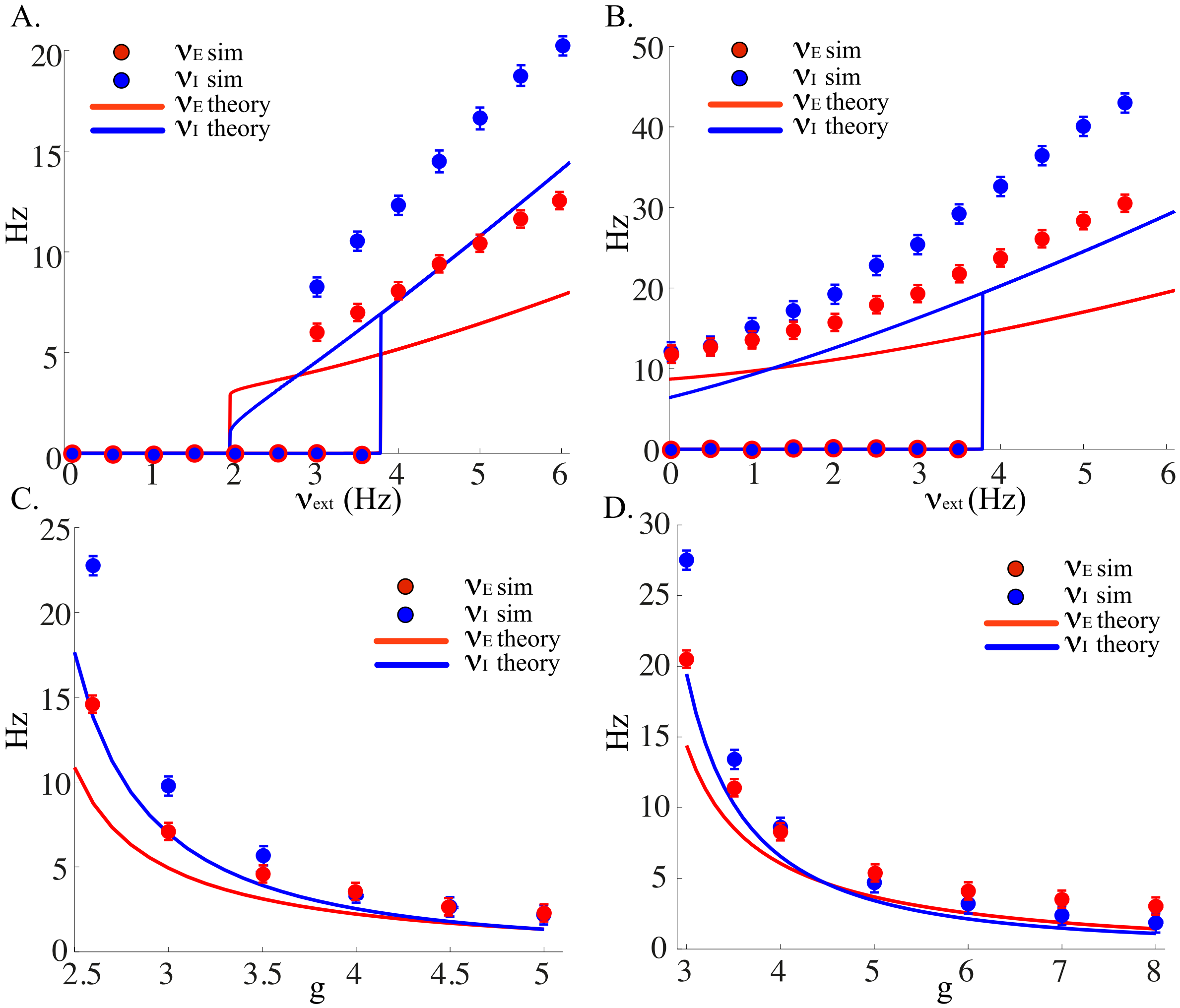

Figure 1: Average excitatory and inhibitory population firing frequency as a function of ν_X, for two values of the E to I synaptic efficacies: $J_{IE} = 0.34$mV (**A.**); $J_{IE} = 0.32$mV (**B.**); for both panels, $J_{EE} = 0.2$mV, $g = 3$, $D_{EE} = 20$ms, $D_{II} = 10$ms. Each simulation point is obtained by averaging the population firing frequency over $T = 10s$. **C., D.** Average excitatory and inhibitory population firing frequency as a function of g for two values of the synaptic efficacies: $J_{IE} = 0.34$mV (**C.**); $J_{IE} = 0.32$mV (**D.**); for both panels, $J_{EE} = 0.2$mV, $\nu_X = 3.8$ (i.e. $\nu_X = 0.76\nu_\theta$), $D_{EE} = 20$ms, $D_{II} = 10$ms. The error bars indicate the standard error of the temporal mean.

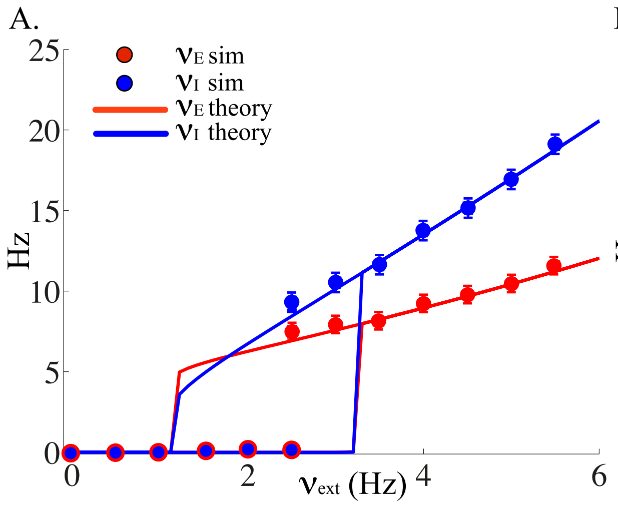

Figure 2: Average excitatory and inhibitory population firing frequency as a function of ν_X; for both panels $J_{EE} = 0.2$mV, $J_{IE} = 0.32$mV, $g = 3$. Each simulation point is obtained by averaging the population firing frequency over $T = 10s$. **A.** $D_{EE} = 10$ms, $D_{II} = 5$ms. **B.** $D_{EE} = 20$ms, $D_{II} = 10$ms. The error bars indicate the standard error over the temporal mean.

shown in Fig. 1. We therefore investigated the source of this discrepancy. A candidate for this discrepancy is the finite amplitude of the synaptic couplings, that potentially lead to deviations from the diffusion approximation, which allows to treat synaptic currents as Gaussian noise (Eq. 3). To understand whether deviations from the diffusion approximation are the source of the observed discrepancies, we replaced the classic Siegert-Ricciardi transfer function (Eq. 7) with the recently obtained transfer function of LIF neurons receiving a combination of excitatory and inhibitory shot noise processes with exponentially distributed amplitudes (Richardson and Swarbrick 2010).

Richardson and Swarbrick (2010) computed the firing rate of a neuron receiving shot noise from pre-synaptic E and I afferents with exponentially distributed amplitudes J_{aE} and J_{aI},

$$\begin{aligned}\nu_a = \Phi_{SN,a}(\nu_E, \nu_I) &= \left(\tau_{m,a} \int_0^{1/J_{aE}} \frac{dc}{c} \frac{1}{Z_a(c, \nu_E, \nu_I)} \left[\frac{e^{cV_\theta}}{1 - J_{aE}c} - e^{cV_R}\right]\right)^{-1}, \\ Z_a(c, \nu_E, \nu_I) &= \frac{1}{(1 - J_{aE}c)^{\tau_{m,a}C_E(\nu_E + \nu_X)}(1 - j_{aI}c)^{\tau_{m,a}C_I\nu_I}},\end{aligned}$$

where $a = E, I$.

The modified theory is then compared to numerical simulations of a network which is identical to the one described in the section “Spiking network model” except for two elements: first, an exponential distribution of synaptic efficacies is implemented; second, the external synaptic current received by a neuron i is no longer given by Eq. 3 as in the standard diffusion approximation, but is rather generated through a Poisson process, in which C_{ext} excitatory neurons send to neuron i synaptic pulses at rate ν_X and with exponentially distributed EPSP

amplitudes. Fig. 2 shows the much better agreement between theory and simulations that can be obtained with such modifications, for the same set of parameters in Fig. 1. This shows that the main factor explaining the discrepancies between theory and simulations in Fig. 1 are the deviations from the diffusion approximation due to the finite size of post-synaptic potentials.

C. Linear stability analysis of the UP-state

Linear stability analysis of networks of LIF neurons with white noise inputs have been described in multiple previous studies of sparsely connected (Brunel and Hakim 1999; Brunel 2000) as well as fully connected networks (Brunel and Hansel 2006; Ledoux and Brunel 2011; Barbieri et al. 2014). Here, we briefly recapitulate the general formalism, and re-express the eigenvalue equation that originally appeared in (Brunel 2000) in terms of neuronal linear response functions. This leads to equations similar to (Ledoux and Brunel 2011), but with an additional contribution of the linear response functions to changes in variance (Lindner and Schimansky-Geier 2001).

The Fokker-Planck equations in the interval $(-\infty, \theta)$ are

$$\tau_a \frac{\partial P_a(V,t)}{\partial t} = \frac{\sigma_a^2(t)}{2} \frac{\partial^2 P_a(V,t)}{\partial V^2} + \frac{\partial}{\partial V}\left[(V - \mu_a(t))P_a(V,t)\right], \tag{3}$$

with

$$\begin{aligned} \mu_a(t) &= C_E J_{aE} \tau_{m,a}\left[\nu_X + \langle \nu_E(t-D)\rangle_{D_{aE}} - \gamma g_a \langle \nu_I(t-D)\rangle_{D_{aI}}\right], && (4)\\ \sigma_a^2(t) &= C_E J_{aE}^2 \tau_{m,a}\left[\nu_X + \langle \nu_E(t-D)\rangle_{D_{aE}} + \gamma g_a^2 \langle \nu_I(t-D)\rangle_{D_{aI}}\right], && (5) \end{aligned}$$

where averages are taken over an exponential distribution of synaptic times

$$\langle \nu_b(t-D)\rangle_{D_{ab}} = \int_0^\infty dD\, \frac{e^{-\frac{D}{D_{ab}}}}{D_{ab}}\, \nu_b(t-D), \tag{6}$$

and $a, b \in \{E, I\}$ for excitatory and inhibitory neurons.

The boundary conditions are

$$\begin{aligned} \frac{\partial P_a}{\partial V}(\theta, t) &= -\frac{2\nu_a(t)\tau_a}{\sigma_a^2(t)}, \\ \frac{\partial P_a}{\partial V}(V_r^+, t) - \frac{\partial P_a}{\partial V}(V_r^-, t) &= -\frac{2\nu_a(t-\tau_{rp})\tau_a}{\sigma_a^2(t)}, && (7)\\ \lim_{V\to-\infty} P_a(V,t) &= 0, \qquad \lim_{V\to-\infty} V P_a(V,t) = 0, \end{aligned}$$

and the normalization

$$\int_{-\infty}^{\theta} P_a(V,t)dV + p_{ra} = 1, \quad p_{ra} = \int_{t-\tau_{rp}}^{t} du\ \nu_a(u). \tag{8}$$

The stationary solutions $P_{a0}(V)$ are given by

$$P_{a0}(V) = \frac{2\nu_{a0}\tau_a}{\sigma_{a0}} exp\left[-\frac{(V-\mu_{a0})^2}{\sigma_{a0}^2}\right]\int_{\frac{V-\mu_{a0}}{\sigma_{a0}}}^{\frac{\theta-\mu_{a0}}{\sigma_{a0}}} du\Theta(u-V_r)e^{u^2}, \tag{9}$$

$$p_{ra0} = \nu_{a0}\tau_{rp}, \tag{10}$$

with

$$\mu_{a0} = C_E J_{aE}\tau_a\left[\nu_X+\nu_{E0}-\gamma g_a\nu_{I0}\right], \tag{11}$$

$$\sigma_{a0}^2 = C_E J_{aE}^2\tau_a\left[\nu_X+\nu_{E0}+\gamma g_a^2\nu_{I0}\right], \tag{12}$$

and

$$\frac{1}{\nu_{a0}} = \tau_{rp} + 2\tau_a\int_{\frac{V-\mu_{a0}}{\sigma_{a0}}}^{\frac{\theta-\mu_{a0}}{\sigma_{a0}}} due^{u^2}\int_{-\infty}^{u} dve^{-v^2}. \tag{13}$$

It is convenient to use reduced variables for the potential

$$y_a = \frac{V-\mu_{a0}}{\sigma_{a0}}, \quad y_{a\theta} = \frac{\theta-\mu_{a0}}{\sigma_{a0}}, \quad y_{ar} = \frac{V_r-\mu_{a0}}{\sigma_{a0}}, \tag{14}$$

for the probability distribution

$$P_a = \frac{2\tau_a\nu_{a0}}{\sigma_{a0}}Q_a, \tag{15}$$

for the rates

$$\frac{1}{\nu_{a0}} = \tau_{rp} + 2\tau_a\int_{y_{ar}}^{y_{a\theta}} due^{u^2}\int_{-\infty}^{u} dve^{-v^2}, \tag{16}$$

$$\nu_a = \nu_{a0}(1+n_a(t)), \tag{17}$$

and for various constants

$$G_{ab} = \frac{C_b J_{ab}\tau_{m,a}\nu_{b0}}{\sigma_{a0}}, \quad H_{ab} = \frac{C_b J_{ab}^2\tau_{m,a}\nu_{b0}}{\sigma_{a0}^2}. \tag{18}$$

The next step is to expand the Fokker-Planck equations about the stationary solutions $Q_{a0}(y_a)$

$$Q_a(y_a,t) = Q_{a0}(y_a) + Q_{a1}(y_a,t) + ... \tag{19}$$

$$n_a(t) = n_{a1}(t) + ... \tag{20}$$

and looking for separable variables solutions

$$Q_{a1}(y_a,t) = \frac{n_{a1}(t)}{n_{a1}(0)}\tilde{Q}_a(y_a), \tag{21}$$

one gets the equations for $n_{a1}(t)$

$$n_{a1}(t) = n_{a1}(0)e^{\lambda t} = \tilde{n}_a(\lambda)e^{\lambda t}, \tag{22}$$

and for $\tilde{Q}_a(y_a)$, depending on the eigenvalues λ.

The general solution of the eigenfunctions equations can be written as a linear combination of two independent solutions of the homogeneous equation, ϕ_1 and ϕ_2,

$$\begin{aligned}
\phi_1(y_a, \lambda) &= M\left[\frac{1-\lambda\tau_a}{2}, \frac{1}{2}, -y_a^2\right], \qquad (23)\\
\phi_2(y_a, \lambda) &= \frac{\sqrt{\pi}}{\Gamma(\frac{1+\lambda\tau_a}{2})}M\left[\frac{1-\lambda\tau_a}{2}, \frac{1}{2}, -y_a^2\right] + \frac{2\sqrt{\pi}y_a}{\Gamma(\frac{\lambda\tau_a}{2})}M\left[1-\frac{\lambda\tau_a}{2}, \frac{3}{2}, -y_a^2\right],
\end{aligned}$$

with $M[a,b,z]$ the confluent hypergeometric function, plus a particular solution of the inhomogeneous one

$$\tilde{Q}_a^p(y_a, \lambda) = \sum_{b=E,I} \tilde{Q}_{ab}^p(y_a, \lambda)\tilde{n}_b(\lambda), \tag{24}$$

$$\tilde{Q}_{ab}^p(y_a, \lambda) = \frac{1}{1+\lambda D_{ab}}\left(\frac{s_b G_{ab}}{1+\lambda\tau_a}\frac{dQ_{a0}}{dy_a} + \frac{H_{ab}}{2(2+\lambda\tau_a)}\frac{d^2Q_{a0}}{dy_a^2}\right). \tag{25}$$

Explicitely

$$\tilde{Q}_a(y_a > y_{ar}, \lambda) = \alpha_a^+(\lambda)\phi_1(y_a, \lambda) + \beta_a^+(\lambda)\phi_2(y_a, \lambda) + \tilde{Q}_a^p(y_a, \lambda), \tag{26}$$

$$\tilde{Q}_a(y_a < y_{ar}, \lambda) = \alpha_a^-(\lambda)\phi_1(y_a, \lambda) + \beta_a^-(\lambda)\phi_2(y_a, \lambda) + \tilde{Q}_a^p(y_a, \lambda). \tag{27}$$

The boundary conditions are eight linear equations that determine the eight coefficients $\alpha_a^+, \alpha_a^-, \beta_a^+, \beta_a^-$.

Further conditions $\alpha_a^- = 0$ are needed in order to obtain an integrable $\tilde{Q}_a(y_a, \lambda)$, and can be derived from the solution of the system of the boundary conditions.

Eventually, one finds that the eigenvalues λ are solutions of the equation

$$A_{EE}(\lambda)A_{II}(\lambda) - A_{IE}(\lambda)A_{EI}(\lambda) = 0, \tag{28}$$

where

$$A_{ab}(\lambda) = [1 + R_{rp,a}(\lambda)]\,\delta_{ab} + \frac{C_b}{1+\lambda D_{ab}}\left[s_b J_{ab} R_{\mu,a}(\lambda) - J_{ab}^2 R_{\sigma^2,a}(\lambda)\right], \tag{29}$$

describes the net effect of the instantaneous firing rate of population b on population a. The variable s_b is defined as $s_E = -1, s_I = 1$. The first term relates to the refractory period τ_{rp}

$$R_{rp,a}(\lambda) = \frac{U(y_{ar}, \lambda\tau_{m,a})(1-e^{-\lambda\tau_{rp}})}{U(y_{a\theta}, \lambda\tau_{m,a}) - U(y_{ar}, \lambda\tau_{m,a})}. \tag{30}$$

The second term in the r.h.s. of Eq. (29) describes the effect of this instantaneous firing rate through the mean synaptic inputs; it is proportional to the linear firing rate response function

$$R_{\mu,a}(\lambda) = \frac{\tau_{m,a}\nu_{b0}}{\sigma_{a0}(1+\lambda\tau_{m,a})}\left[\frac{\frac{\partial U}{\partial y}(y_{a\theta}, \lambda\tau_{m,a}) - \frac{\partial U}{\partial y}(y_{ar}, \lambda\tau_{m,a})}{U(y_{a\theta}, \lambda\tau_{m,a}) - U(y_{ar}, \lambda\tau_{m,a})}\right], \tag{31}$$

where $U(y, \lambda) = e^{y^2}\ \phi_2(y, \lambda)$.

The third term in the r.h.s. of Eq. (29) describes the effect of this instantaneous firing rate through the variance of synaptic inputs; it is proportional to another linear response function,

$$R_{\sigma^2,a}(\lambda) = \frac{\tau_{m,a}\nu_{b0}}{\sigma_{a0}^2(2 + \lambda\tau_{m,a})}\left[\lambda\tau_{m,a} + \frac{y_{a\theta}\frac{\partial U}{\partial y}(y_{a\theta}, \lambda\tau_{m,a}) - y_{ar}\frac{\partial U}{\partial y}(y_{ar}, \lambda\tau_{m,a})}{U(y_{a\theta}, \lambda\tau_{m,a}) - U(y_{ar}, \lambda\tau_{m,a})}\right]. \qquad (32)$$

Note that this term disappears in networks in which the synaptic couplings scale as $1/C$, in the large C limit. To investigate the effect of variance dynamics on the instabilities of the asynchronous state, we set $R_{\sigma^2,a} = 0$ in Eq. (29).

Note that in the strong noise, low firing rate regime investigated in this paper $R_\mu(f)$ is a monotonically decreasing function of f, which is essentially constant below a cut-off frequency which is of order $1/\tau_m$ at low frequencies (Ostojic and Brunel 2011), and then decay asymptotically to zero as $1/\sqrt{f}$ (Brunel and Hakim 1999; Lindner and Schimansky-Geier 2001; Brunel et al. 2001). On the other hand, R_{σ^2} has a finite limit when f goes to infinity (Lindner and Schimansky-Geier 2001).

D. Treatment of firing rate adaptation

Following La Camera et al. (2004), we replace adaptation currents by their mean value $\beta\nu_{E0}$. The mean-field equations (6-9) are unaffected, except that the equation for μ_{E0} is replaced by

$$\mu_{E0} = \tau_{mE}C_E J_{EE}(\nu_X + \nu_{E0} - \gamma g_E \nu_{I0}) - \beta\nu_{E0}$$

E. Spiking Network Model with exponentially decaying synaptic currents

As before, the total current arriving to a postsynaptic neuron is due to the activity of its local (pre-synaptic) afferents and to the current elicited by external inputs.

$$I_i(t) = I_i^{ext}(t) + I_i^{rec}(t) \qquad (33)$$

where $I_i^{ext}(t)$ is the external input current and $I_i^{rec}(t)$ is the total recurrent synaptic current.

We now model the recurrent contribution to the post-synaptic current as the sum of contribution that are mediated by NMDA, AMPA and GABA receptors with distinct time constants. The total recurrent currents become

$$I_i^{rec}(t) = I_i^N(t) + I_i^{AM}(t) - I_i^G(t) \qquad (34)$$

where each of the synaptic currents is described by a separate differential equation

$$\tau_N \dot{I}_{i,a}^N(t) = -I_{i,a}^N + x_{E,I} I_{i,a}^E(t) \qquad (35)$$

$$\tau_{AM} \dot{I}_{i,a}^{AM}(t) = -I_{i,a}^{AM} + (1 - x_{E,I}) I_{i,a}^E(t) \qquad (36)$$

$$\tau_G \dot{I}_{i,a}^G(t) = -I_{i,a}^G + I_{i,a}^I(t) \qquad (37)$$

where

$$
\begin{aligned}
I_{i,a}^{E}(t) &= \tau_{m,a}\sum_{j=1}^{N_E} c_{ij,aE} J_{aE} \sum_k \delta(t - t_{j,E}^k - D_{ij,aE}) && (38)\\
I_{i,a}^{I}(t) &= \tau_{m,a}\sum_{j=1}^{N_I} c_{ij,aI} J_{aI} \sum_k \delta(t - t_{j,I}^k - D_{ij,aI}), && (39)
\end{aligned}
$$

$x_{E,I}$ is the fraction of the charge coming from excitatory afferents which is mediated by NMDA receptors, which mediates a slow post-synaptic current. The remaining fraction of the charge $(1 - x_{E,I})$ is mediated by AMPA receptors and elicits a faster current. The current coming from inhibitory afferents is mediated by GABA receptors. Upon arrival of a pre-synaptic spike at time t_k, the postsynaptic current instantaneously receives a "kick" proportional to the synaptic efficiency J (in mV), followed by an exponential decay with time constant τ_{syn}, where $\tau_{syn} = \tau_{AM,N,G}$. In what follows we set x_I, as well as the synaptic delays to zero.

F. Spiking Network Model with conductance-based synaptic currents

We simulated a network with conductance-based synapses in which the recurrent synaptic currents of neuron i in population a $(= E, I)$ are given by

$$
I_{i,a}^{rec}(t) = (V_E - V)s_{i,a}^{E}(t) + (V_I - V)s_{i,a}^{I}(t) \qquad (40)
$$

where $V_E = 70$mV is the excitatory reversal potential, $V_I = 0$mV is the inhibitory reversal potential, and $s_{i,a}^{E,I}(t)$ represent excitatory and inhibitory synaptic conductances (normalized by the leak conductance), which evolve in time according to:

$$
\tau_b \dot{s}_{ia}^{b}(t) = -s_{ia}^{b} + \tau_{m,a}\sum_{j=1}^{N_b} c_{ij,ab} g_{ab} \sum_k \delta(t - t_{j,b}^k) \qquad (41)
$$

where τ_b $(b = E, I)$ is the decay time constant of E/I synaptic currents, and g_{ab} is the (normalized) conductance of b synapses onto a neurons. The conductances were obtained by dividing the synaptic efficacies in the current-based network by the driving force at threshold, i.e. $(V_E - \theta)$ for E synapses, $(V_I - \theta)$ for I synapses, and by rescaling E synapses by an additional factor γ. Excitatory neurons were also endowed with an adaptation current described by Eqs. (10,11) in the main text.

G. Identification of transitions between up and down states in simulations

Up and down state transitions were identified using the same method described in Neske et al. (2015). In particular, both a fast and a slow exponential moving average were calculated over the excitatory population firing rate. The intersection points of the two moving averages determined onset and offset of a state transition.

References

Barbieri, F., A. Mazzoni, N. K. Logothetis, S. Panzeri, and N. Brunel (2014). Stimulus dependence of local field potential spectra: experiment versus theory. *J. Neurosci. 34*, 14589–14605.

Brunel, N. (2000). Dynamics of sparsely connected networks of excitatory and inhibitory spiking neurons. *J. Comput. Neurosci. 8*, 183–208.

Brunel, N., F. Chance, N. Fourcaud, and L. Abbott (2001). Effects of synaptic noise and filtering on the frequency response of spiking neurons. *Phys. Rev. Lett. 86*, 2186–2189.

Brunel, N. and V. Hakim (1999). Fast global oscillations in networks of integrate-and-fire neurons with low firing rates. *Neural Comp. 11*, 1621–1671.

Brunel, N. and D. Hansel (2006). How noise affects the synchronization properties of networks of inhibitory neurons. *Neural Comp. 18*, 1066–1110.

La Camera, G., A. Rauch, H. R. Luscher, W. Penn, and S. Fusi (2004). Minimal models of adapted neuronal responses to in-vivo like input currents. *Neural Comp. 16*, 2101–2124.

Ledoux, E. and N. Brunel (2011). Dynamics of networks of excitatory and inhibitory neurons in response to time-dependent inputs. *Front Comput Neurosci 5*, 25.

Lindner, B. and L. Schimansky-Geier (2001). Transmission of noise coded versus additive signals through a neuronal ensemble. *Phys. Rev. Lett. 86*, 2934–2937.

Neske, G. T., S. L. Patrick, and B. W. Connors (2015). Contributions of diverse excitatory and inhibitory neurons to recurrent network activity in cerebral cortex. *J. Neurosci. 35*, 1089–1105.

Ostojic, S. and N. Brunel (2011). From spiking neuron models to linear-nonlinear models. *PLOS Comp Biol 7*, e1001056.

Richardson, M. J. and R. Swarbrick (2010). Firing-rate response of a neuron receiving excitatory and inhibitory synaptic shot noise. *Phys. Rev. Lett. 105*, 178102.
